# Spatiotemporal Analysis of Hepatitis C Virus Infection

**DOI:** 10.1371/journal.ppat.1004758

**Published:** 2015-03-30

**Authors:** Ana Shulla, Glenn Randall

**Affiliations:** Department of Microbiology, The University of Chicago, Chicago, Illinois, United States of America; The Scripps Research Institute, UNITED STATES

## Abstract

Hepatitis C virus (HCV) entry, translation, replication, and assembly occur with defined kinetics in distinct subcellular compartments. It is unclear how HCV spatially and temporally regulates these events within the host cell to coordinate its infection. We have developed a single molecule RNA detection assay that facilitates the simultaneous visualization of HCV (+) and (−) RNA strands at the single cell level using high-resolution confocal microscopy. We detect (+) strand RNAs as early as 2 hours post-infection and (−) strand RNAs as early as 4 hours post-infection. Single cell levels of (+) and (−) RNA vary considerably with an average (+):(−) RNA ratio of 10 and a range from 1–35. We next developed microscopic assays to identify HCV (+) and (−) RNAs associated with actively translating ribosomes, replication, virion assembly and intracellular virions. (+) RNAs display a defined temporal kinetics, with the majority of (+) RNAs associated with actively translating ribosomes at early times of infection, followed by a shift to replication and then virion assembly. (−) RNAs have a strong colocalization with NS5A, but not NS3, at early time points that correlate with replication compartment formation. At later times, only ~30% of the replication complexes appear to be active at a given time, as defined by (−) strand colocalization with either (+) RNA, NS3, or NS5A. While both (+) and (−) RNAs colocalize with the viral proteins NS3 and NS5A, only the plus strand preferentially colocalizes with the viral envelope E2 protein. These results suggest a defined spatiotemporal regulation of HCV infection with highly varied replication efficiencies at the single cell level. This approach can be applicable to all plus strand RNA viruses and enables unprecedented sensitivity for studying early events in the viral life cycle.

## Author Summary

The stages of the viral life cycle are spatially and temporally regulated to coordinate the infectious process in a way that maximizes successful replication and spread. In this study, we used RNA in situ hybridization (ISH) to simultaneously detect HCV (+) and (−) RNAs and analyze the kinetics of HCV infection at the single cell level as well as visualize HCV RNAs associated with actively translating ribosomes, markers of viral replication compartment formation, active RNA replication, nucleocapsid assembly, and intracellular virions. We observed a spatial linkage between sites of viral translation and replication, in addition to replication and assembly. HCV (+) RNAs follow a tight temporal regulation. They are initially associated with translating ribosomes, followed by a peak of replication that achieves a steady state level. The remaining HCV (+) RNAs are then devoted to virion assembly. Analysis of HCV (−) RNAs revealed that low levels of transient RNA replication occur early after infection prior to the formation of devoted replication compartments and robust replication. This suggests that HCV synthesizes additional (+) and (−) strands early in infection, likely to decrease its reliance on maintaining the integrity of the initially infecting (+) RNA.

## Introduction

Hepatitis C virus (HCV) belongs to the *Flaviviridae* family of enveloped, positive-stranded RNA viruses. Following productive entry into hepatocytes, the 9.6 kb HCV genome is translated to produce a single large polyprotein [1], which is cleaved by viral and host proteases to yield ten distinct protein products [2]. These proteins include the structural proteins (core, E1 and E2) and the non-structural proteins (p7, NS2, NS3, NS4A, NS4B, NS5A, and NS5B). The five “replicase” proteins NS3 to NS5B are essential and sufficient for HCV RNA replication [3,4]. Similar to all other positive strand RNA viruses, HCV induces rearrangements of intracellular membranes to create a favorable microenvironment for RNA replication to occur [5–8]. Replication complex formation appears to require the viral NS4B and NS5A proteins [5,9]. NS5B, the viral RNA-dependent RNA polymerase is the key enzyme of the replicase complex [10,11]. Using the (+) strand genome as a template, NS5B first synthesizes a complementary (−) strand, resulting in a double-stranded (ds) RNA intermediate, and then proceeds to transcribing progeny (+) strands. Newly synthesized (+) strand RNAs are then thought to be shuttled out of replication compartments to serve as templates for further translation by cellular ribosomes or become encapsidated into assembling virions on the surface of lipid droplets (LDs) [12]. Although these processes are likely linked, a single viral (+) strand RNA can only be involved in either translation, replication or packaging at a given time, and the switch from one process to another has to be regulated [13]. For HCV, the switch from translation to replication is unclear. The cellular protein Ewing sarcoma breakpoint region 1 (EWSR1) binds to the viral RNA cis acting replication element (CRE), and has been proposed to regulate the switch from translation to replication by modulating the kissing interaction between the CRE and a RNA stem-loop structure in the HCV 3’ UTR [14]. Similarly, for polioviruses, the switch from translation to replication is regulated by the action of viral proteases on a cellular protein binding to the 5’cloverleaf viral RNA structure [15]. The switch from replication to assembly is not well understood, however it has been suggested that the phosphorylation state of NS5A might regulate the process [16]. It is also possible that HCV (+) RNA fate is spatially regulated by the distinct subcellular localizations of translation, replication and assembly.

It is not clear how HCV spatially and temporally regulate its lifecycle within the host cell. Mathematical models have been developed to study HCV RNA dynamics during primary infection of chimpanzees [17], and humans (liver transplantation [18,19], response to IFN [20] and ribavirin treatment [21]), in addition to HCV replicons in cell culture [22–25]. Recently, data on the dynamics of infectious HCV RNA in cell culture became available [26]. While these past studies were focused on whole cell populations, organs and organisms, data on viral RNA kinetics in single cells are lacking. Advances in single RNA detection methods using ISH followed by amplification [27] enable the analysis of HCV RNA at the single cell level. Previous studies have utilized this approach to detect (+) strand HCV RNAs in hepatoma cell lines [28] as well as in infected human liver biopsies [29]. To better understand the spatiotemporal organization of HCV infection, we have developed RNA detection methods that allow for simultaneous visualization of HCV (+) and (−) RNA strands at the single cell level. We have combined this approach with 4-color high-resolution confocal microscopy to study the localization of (+) and (−) HCV RNAs with viral and host proteins of interest.

Using this single cell RNA detection approach, we can interrogate the cell-to-cell variability of HCV infection kinetics. Moreover, we can determine whether (+) strand fates are regulated temporally. The approach developed in this study can be generically applicable to all RNA viruses and enables unprecedented sensitivity for studying early events in the viral life cycle, which are unappreciated for viruses of relatively low replicative capacity, including some viruses of the *Flaviviridae*, due to limits of detection.

## Results

### Specificity of HCV RNA labeling

**Fig 1:**
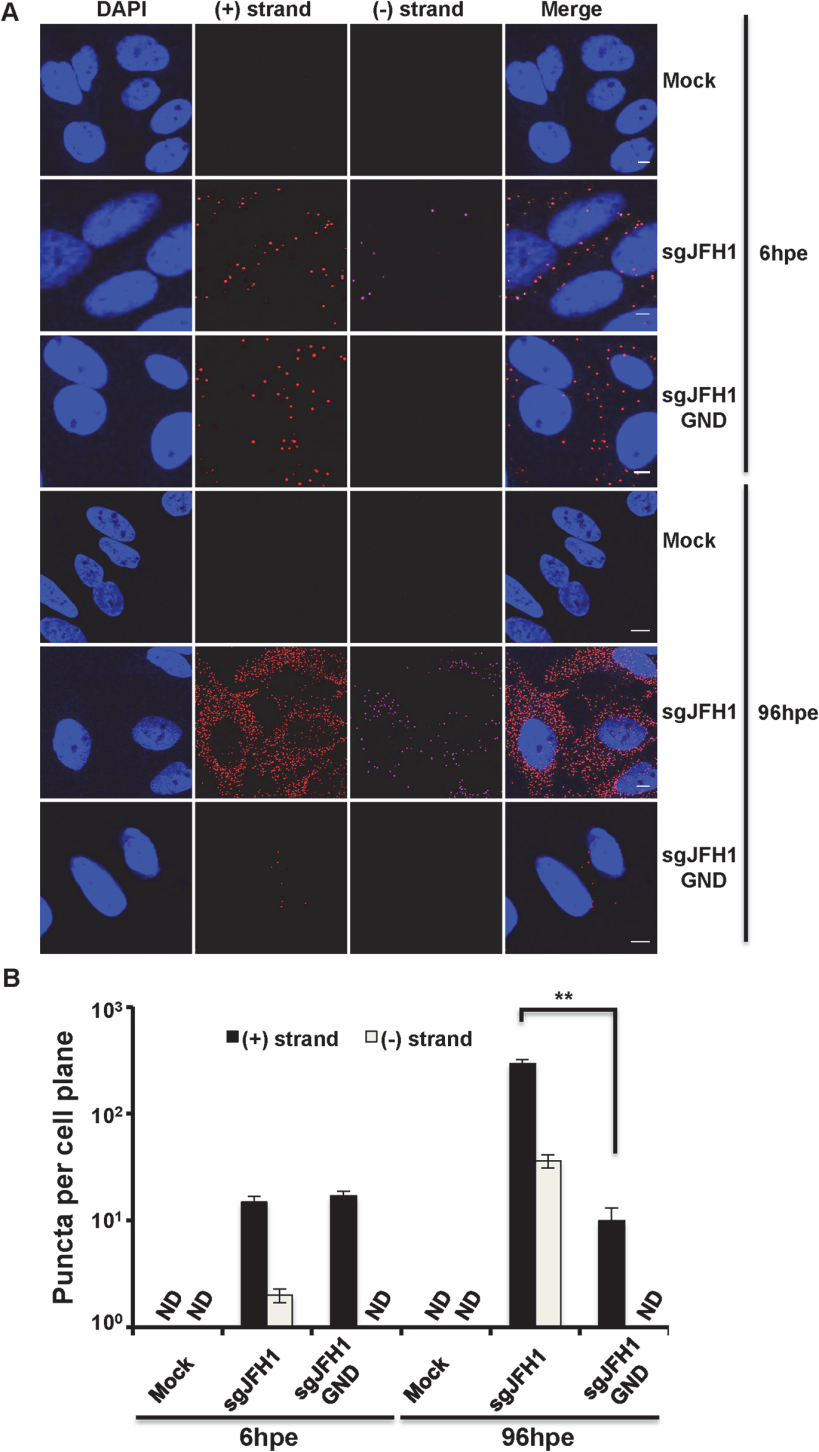
Strand specific HCV RNA detection in fixed cells. **A**. Huh-7.5 cells were electroporated with the indicated HCV RNA constructs and cells were fixed and processed for RNA detection at 6 and 96 hours post-electroporation. Scale bar is 5 μm. **B**. Quantification of images in panel A. Error bars represent standard deviation from 25 different images. ND = not detected. ** p = 0.0008.

We used the QuantiGene ViewRNA ISH detection system to specifically detect HCV (+) and (−) strand RNAs in single cells. Briefly, non-overlapping oligonucleotide probe sets specific to either the (+) or (−) strand of HCV NS3/4A region were hybridized to target RNAs and sequential hybridization steps provided up to 8,000-fold signal amplification. To validate the specificity of labeling, wild type or polymerase defective (GND) subgenomic JFH1 HCV replicon RNAs were electroporated into Huh-7.5 cells, which were then fixed and processed for HCV RNA detection (Fig. 1A) and quantification (Fig. 1B). (+) strand RNA, comprising a mix of input and newly synthesized RNA, was abundant at 6 hours post-electroporation (hpe) and increased up to 20 fold by 96 hpe for the wild type, but not the polymerase defective replicon containing cells. (−) strand RNA was also detected at 6 hpe and increased at a similar rate as the (+) strand by 96 hpe for wild type HCV. There was no (−) strand detected at any of the time points for the GND replicon, thus confirming that polymerase function is required for (−) strand RNA detection. As expected, there was no signal for either (+) or (−) HCV RNAs detected in mock infected cells.

To further confirm the specificity of our (−) strand RNA labeling in the context of HCV infection, we used the HCV direct-acting antivirals Sofosbuvir and Daclatasvir. Sofosbuvir is a nucleotide analogue inhibitor of the NS5B polymerase [30] already approved for anti-HCV therapy in combination with ribavirin or other direct acting antivirals such as Daclatasvir, Ledispavir, or Simeprevir [31]. Daclatasvir is a potent inhibitor of the NS5A protein [32] that is already approved for anti-HCV therapy in Europe and Japan [33]. As expected, there was very little (−) strand accumulation at 6 and 48 hours post-infection (hpi) in cells treated with either Sofosbuvir or Daclatasvir at time of infection. In contrast, (−) strand was detected at 6 hpi and increased 10-fold by 48 hpi in DMSO treated cells (S1 Fig.). We did not observe an effect of either inhibitor on (+) strand levels at 6 hpi, however by 48 hpi there was a drastic decrease in (+) strand numbers due to the replication defect imposed by the antiviral drugs.

### Kinetics of HCV RNA accumulation

**Fig 2:**
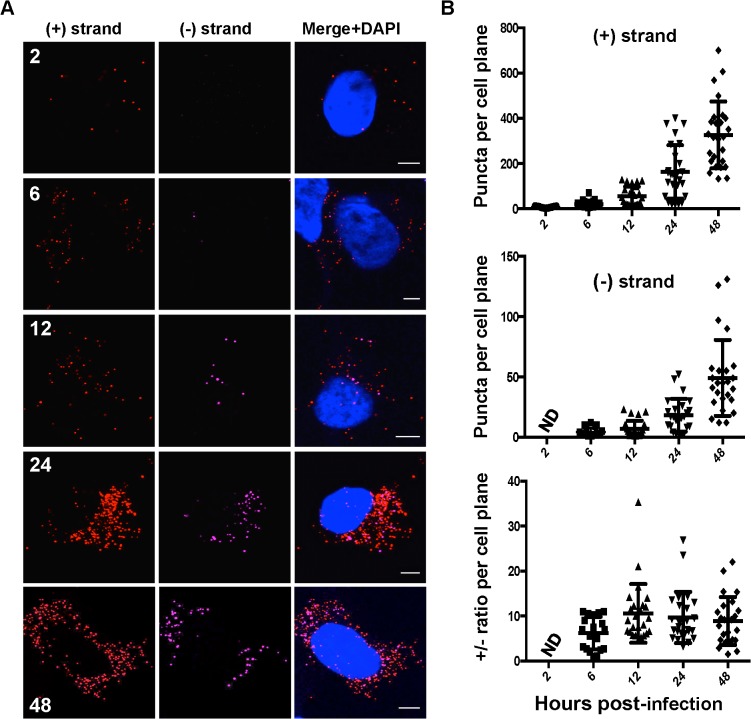
Kinetics of HCV RNA accumulation during infection. **A**. Huh-7.5 cells were infected with HCV at MOI = 1.5 and at the indicated times post-infection cells were fixed and processed for strand specific RNA detection. Scale bar is 5 μm. **B**. Individual (+) and (−) strand puncta for each time point were quantified and graphed using Prism software.

The kinetics of accumulation and ratios of HCV (+) and (−) strand RNAs are unknown at the single cell level. To this end, we quantified HCV (+) and (−) strand RNAs at the single cell level over a time course of infection. As expected, we observed (+) and (−) strand HCV RNA numbers increased over time (Fig. 2A). Quantitation of (+) and (−) strand RNAs showed considerable cell-to-cell variability (>10-fold) with respect to the number of RNA puncta per cell plane (Fig. 2B). (+) strand puncta could be detected as early as 2 hpi in ~80% of cells, which likely reflected input genomic RNA. (−) strand puncta were reliably detected in cells at 6 hpi, suggesting that viral RNA replication is established by 6 hpi. Consistent with this interpretation, we observe modest increases in (+) RNA at 6 and 12 hpi. Much larger increases in (+) and (−) RNA accumulation occurred between 12 and 24 hpi, suggesting that this time point correlates with more robust HCV replication. By 48 hpi, an average of 331±52 (+) RNAs and 49±12 (−) RNAs were observed per cell plane. In order to correlate this value with HCV RNAs per cell, we performed Z-stack analysis of HCV infected cells at 48 hpi and found that the average per cell number was 415±39 (+) RNAs and 94±11 (−) RNAs per cells (S1 Movie). Thus, the fluorescence per cell plane captures the majority of the whole cell RNAs. This high percentage reflects the high fluorescence of the RNA ISH signal, in addition to the relative flatness of Huh-7.5 cells.

We observed a (+) strand to (−) strand ratio of about 10:1 throughout most of the time course, which is in agreement with the ratio determined in infected hepatocytes in humans [34] as well as previous reports using subgenomic replicon systems [3,35]. Notably, this ratio was about 6:1 early in infection (6 hpi), which correlates with the ratio of (+):(−) strands inside replication complexes [35]. Although the average (+):(−) RNA ratio of ~10 was relatively constant, there was significant cell-to-cell variability in ratio, ranging from 1–35.

### Localization of HCV RNAs with active translating ribosomes

**Fig 3:**
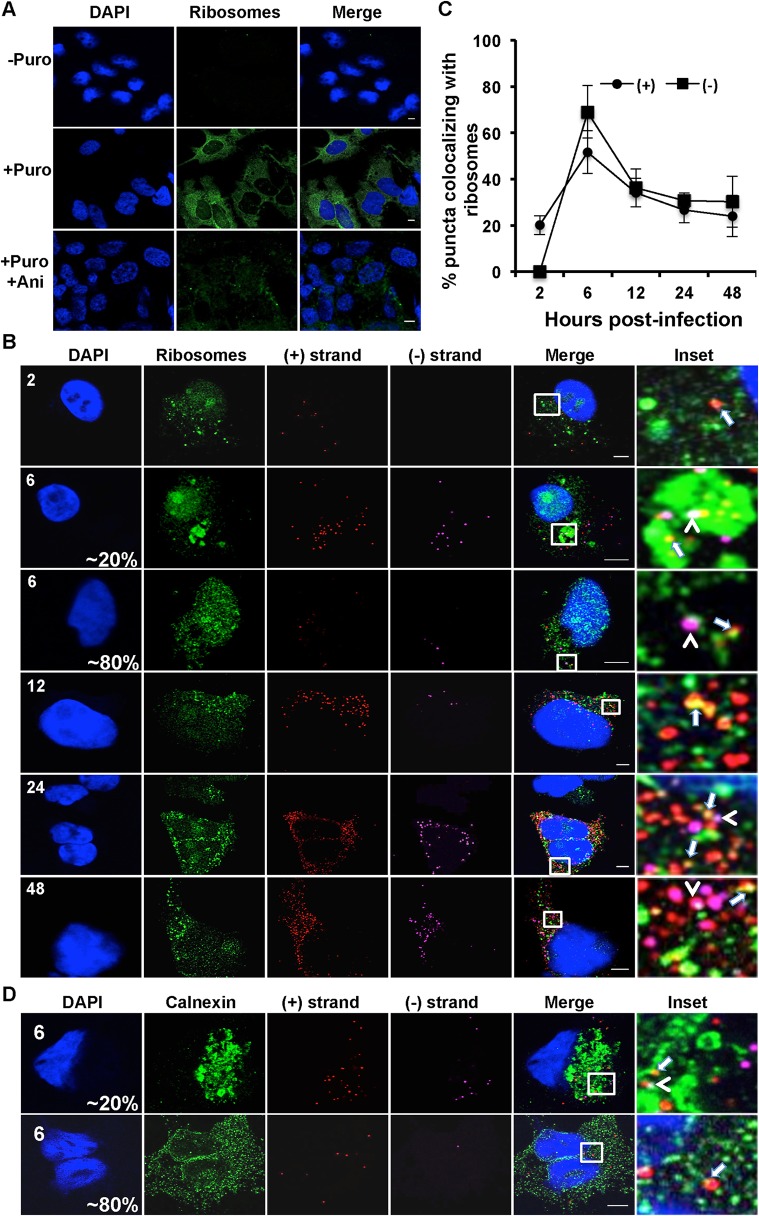
Colocalization of (+) and (−) strand HCV RNAs with active translating ribosomes. **A**. Huh-7.5 cells were infected with HCV at MOI = 1.5 and at 48 hours post-infection cells were left untreated or pre-treated with anisomycin (Ani) (competitive inhibitor of puromycin; 9.4 uM) followed by puromycin (Puro) labeling and digitonin extraction before fixation. Cells were processed for immunofluorescence using the anti-puromycin PMY-2A4 monoclonal antibody. Scale bar is 5 μm. **B**. Huh-7.5 cells were infected with HCV at MOI = 1.5 and at the indicated times post-infection the cells were fixed and processed for strand specific RNA detection followed by immunofluorescence staining for puromycylated ribosomes. Scale bar is 5 μm. Insets represent 10 times magnification of the merged image. Solid arrows point to (+) strand RNA colocalizing with ribosomes; arrowheads point to (−) strand RNA colocalizing with ribosomes. **C**. Quantitation of % colocalization in (B). Each error bar indicates standard deviation from 25 different images. **D**. Huh-7.5 cells were infected with HCV at MOI = 1.5 and at 6 hpi the cells were fixed and processed for strand specific RNA detection followed by immunofluorescence staining for calnexin.

**Fig 4:**
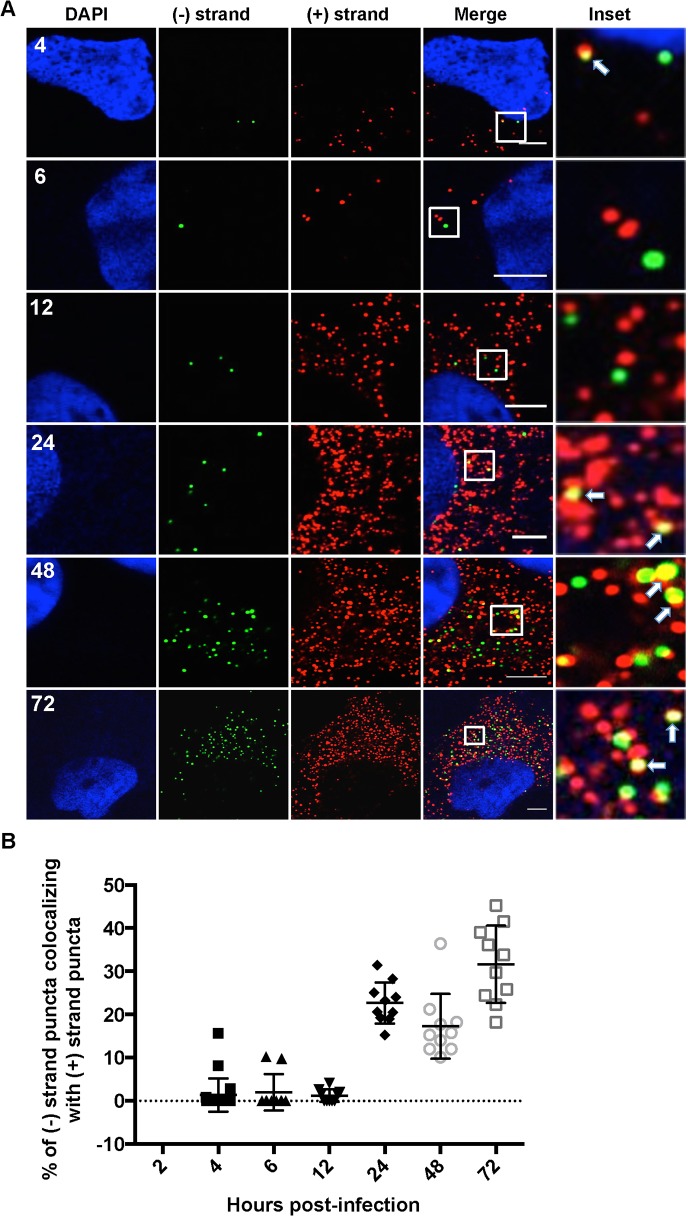
Colocalization of (−) and (+) HCV RNA strands. **A**. Huh-7.5 cells were infected with HCV at MOI = 1.5 and at the indicated times post infection cells were fixed and processed for strand specific RNA detection. Scale bar is 5 μm. Solid arrows point to (+) strand RNA colocalizing with (−) strand RNA. **B**. Quantitation of % colocalization in (A).

**Fig 5:**
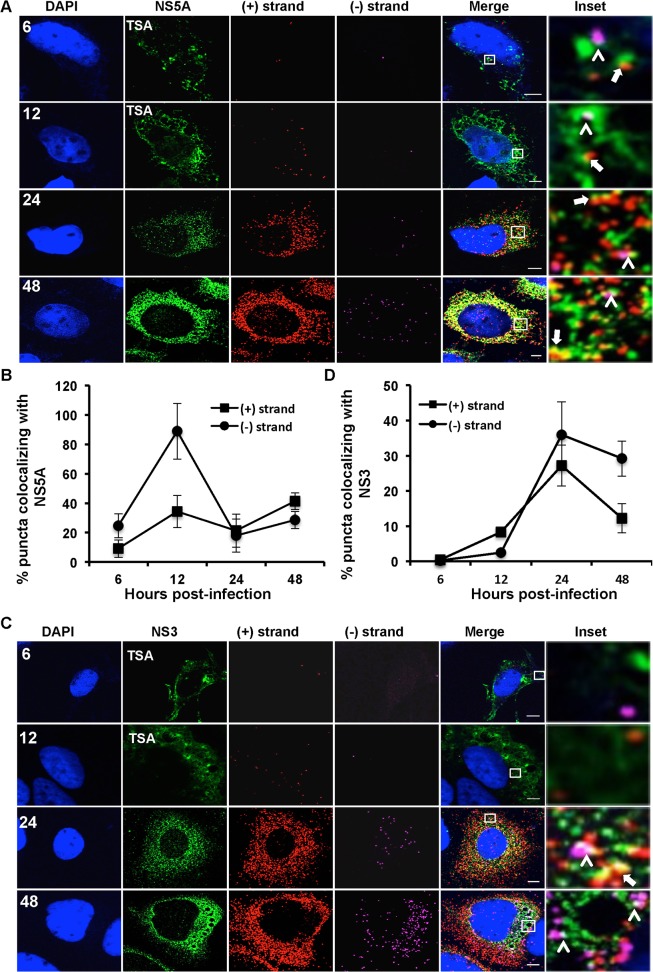
Colocalization of (+) and (−) strand HCV RNAs with NS5A and NS3. Huh-7.5 cells were infected with HCV at MOI = 1.5 and at the indicated times post-infection the cells were fixed and processed for strand specific RNA detection followed by immunofluorescence staining for **A**. NS5A or **C**. NS3. For 6 and 12 hpi samples, antibody signal was amplified using the tyramide signal amplification kit (TSA) as described in the materials and methods section. Scale bar is 5 μm. **B**. Quantitation of (A) **D**. Quantitation of (C). Each error bar indicates standard deviation from 25 different images. Scale bar is 5 μm. Insets represent 10 times magnification of the merged image. Solid arrows point to (+) strand RNA colocalizing with NS5A and NS3; arrowheads point to (−) strand RNA colocalizing with NS5A and NS3.

**Fig 6:**
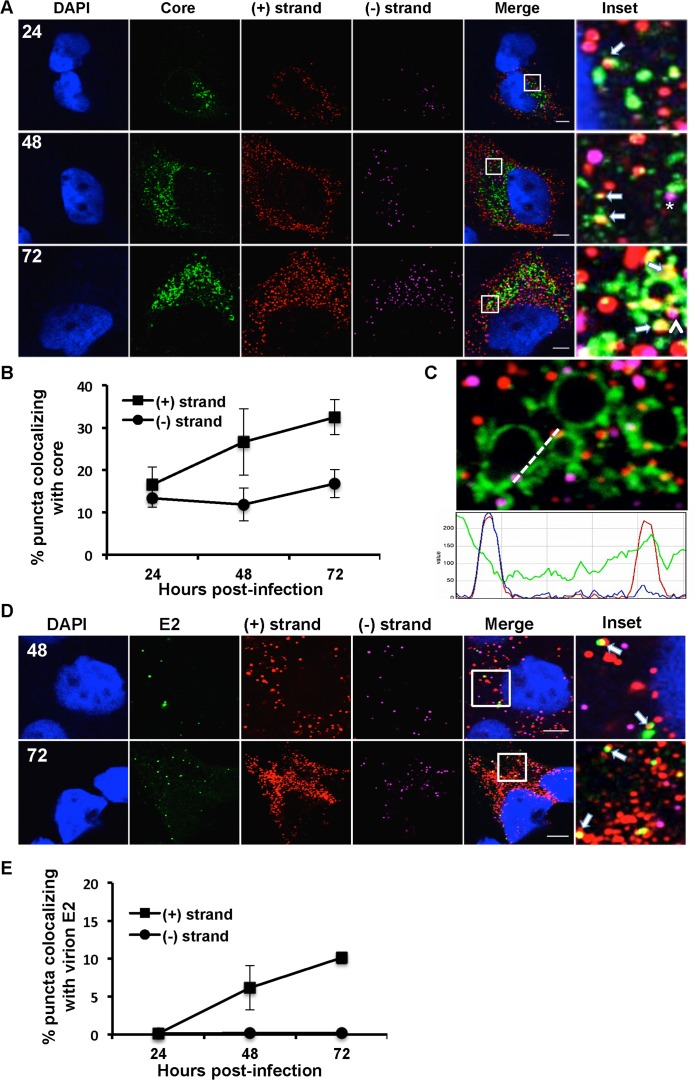
Colocalization of (+) and (−) strand HCV RNAs with core protein and virion E2. **A**. Huh-7.5 cells were infected with HCV at MOI = 1.5, fixed at the indicated times post-infection and processed for strand specific RNA detection followed by immunofluorescence staining for core. Scale bar is 5 um. Solid arrows indicate (+) strand (red) colocalization; arrowheads indicate (−) strand (magenta) colocalization with core (green). Insets represent 10 times magnification of the merged image. The asterisk indicates juxtaposition of (−) strand with core. **B**. Quantification of images in panel A. **C**. A merged image of core colocalization with (+) and (−) HCV RNAs at 48 hpi is shown together with an ImageJ color intensity plot for the white line drawn in the merged image. **D, E**. Huh-7.5 cells were infected with HCV at MOI = 1.5 and at 48 and 72 hpi the cells were fixed and processed for strand specific RNA detection followed by immunofluorescence staining for E2 protein using CBH-5 antibody and quantified. Insets represent 10 times magnification of the merged image.

We next developed a series of microscopy assays to visualize the association of HCV RNA with markers of specific stages of the viral life cycle. HCV translation was defined as the colocalization of (+) RNAs with actively translating ribosomes (Fig. 3). Active RNA replication was defined as colocalization of HCV (−) RNA with (+) RNA or replicase components NS3 and NS5A (Fig. 4, Fig. 5). HCV assembly was defined as colocalization of (+) RNA with core and intracellular virions were defined as colocalization of (+) RNA with virion E2 (Fig. 6). A caveat to the interpretation of these assays is that colocalization of HCV RNA with these markers does not define a physical interaction.

(+) RNA viruses cannot be simultaneously translated (with ribosomes moving 5’ to 3’) and replicated (with the replicase moving 3’ to 5’) [13]. These processes need to be coordinately regulated, either by spatially separating (+) strand RNAs destined for translation and replication, or by regulating the initiation of these processes. In the case of HCV, the mode of regulation is not yet known, nor is it known whether translation occurs in the proximity of replication compartments. To address these questions, we applied a ribosome-bound nascent chain puromycylation assay [36] that detects actively translating ribosomes in our single molecule HCV RNA detection assay. Briefly, puromycin, a Tyr-tRNA mimetic, enters the ribosome A site and terminates translation. Puromycylated nascent chains are stalled on ribosomes by cycloheximide, a chain elongation inhibitor, and detected via fixed cell immunofluorescence using an anti-puromycin monoclonal antibody. In the absence of puromycin, we detect no fluorescent signal, while puromycin labeling is readily detected in the presence of puromycin (Fig. 3A), primarily in the cytoplasm but also some in the nucleus, consistent with a previous report [36]. To confirm the specificity of puromycin labeling, cells were pre-treated with anisomycin, a competitive inhibitor of translation, prior to puromycylation. As expected, anisomycin drastically decreased puromycin labeling (Fig. 3A).

We next performed the puromycylation assay in combination with RNA ISH over a time course of HCV infection (Fig. 3B,C). (+) RNA translation, as defined by (+) RNA localization with puromycylated ribosomes, occurred as early as 2 hpi, with a peak in the percent of (+) RNAs undergoing translation (70%) at 6 hpi. Between 12 and 24 hpi, a steady state level of translated (+) RNAs was achieved at ~35%. While the (−) RNA is unlikely to be associated with actively translating ribosomes, we do observe that a high percentage of HCV (−) RNAs also colocalize in the proximity of puromycylated ribosomes. This indicates that sites of viral RNA replication and translation may be in close proximity. Interestingly, we observed an unusual puromycylation staining pattern in ~20% of the cells at 6 hpi, in which enlarged puromycylation puncta were detected (Fig. 3B). It is possible that these enlarged puncta reflect ER rearrangements (Fig. 3B). In support of this interpretation, we observed a similar localization pattern of the ER marker calnexin at this time point of infection (Fig. 3D).

### Localization of HCV RNAs with replication complex markers

HCV RNA synthesis involves a dsRNA intermediate, which localizes inside the viral induced replication complexes [9,37]. We next quantified (+) and (−) RNA colocalization over a time course of infection as a marker of HCV RNA replication (Fig. 4A,B). We observed that (−) strand puncta colocalizing with (+) strand puncta became detectable as early as 4 hpi; however, it was generally a low frequency event from 4–12 hpi. The frequency of HCV (+) and (−) RNA colocalization increased significantly between 12 and 24 hpi, with ~25–35% of (−) strand puncta colocalized with (+) strand puncta. This percentage remained relatively constant from 24–72 hpi, suggesting that only ~1/3 of (−) strands are actively engaged in RNA replication at a given time. Unfortunately, attempts to correlate the (+) and (−) RNA colocalization with dsRNA localization using the commonly used J2 dsRNA antibody were unsuccessful, due to incompatible fixation and permeabilization conditions.

We next examined the localization of HCV RNAs with viral protein components of the replication complex. HCV NS5A is a multifunctional protein involved in both the replication and assembly stages of HCV life cycle [4,38–40]. NS5A appears to have at least two functions in HCV RNA replication. It is part of the replicase complex that binds viral RNA [3,4] and it also promotes the formation of double membrane vesicles, which are thought to be the sites of viral RNA replication [9]. The function of NS5A in replication complex formation is regulated in part by the interaction of NS5A with its host cofactor, phosphatidylinositol-4-kinase III-α (PI4KA) [41–43]. Additionally, NS5A partially colocalizes with core protein on surface of lipid droplets and is required for virion assembly [12,38]. We performed a time course of (+) and (−) strand RNA colocalization with the NS5A protein during HCV infection (Fig. 5A). Since NS5A protein levels early in infection are undetectable by standard immunofluorescence analysis we used a tyramide signal amplification system (TSA) to detect NS5A at 6 and 12 hpi. We observed limited colocalization of NS5A with either (+) or (−) RNA at 6 hpi; however, there was a dramatic, specific increase in NS5A colocalization with (−) RNA at 12 hpi (~90% of (−) RNA colocalized with NS5A, Fig. 5B). At later time points, ~30% of NS5A colocalized with (−) RNA, which would suggest, only ~30% of (−) strand RNAs undergo replication at later times of infection, which is consistent with the observed colocalization of (−) with (+) RNA. The localization of NS5A with (+) RNA increased over time, consistent with its role in virion assembly (Fig. 5B).

NS3 is also part of the replicase complex and its helicase activity is required for HCV replication and possibly assembly [7,44,45]. In contrast to NS5A, NS3 did not preferentially colocalize with (−) strands at early time points (Fig. 5C,D). At later time points, we observed that NS3 had similar levels of RNA colocalization as was observed for NS5A (~30%). The distinct frequencies of (−) RNA localization with NS3 and NS5A are consistent with the interpretation that NS5A, but not NS3, is involved in the initial formation of replication compartments, while both NS3 and NS5A are involved in replicase function.

### Localization of HCV RNAs with core and E2

The current model for HCV assembly is that ER-derived HCV replication complexes are in close proximity to the sites of virion assembly, intracellular lipid droplets (LDs). The viral core (capsid) protein accumulates on or near the surface of LDs and mutants that attenuate virion assembly lead to a hyper-accumulation of core at the LD [6,46,47]. Viral RNA containing capsids are then enveloped at the ER and egress cells via the secretory pathway in association with components of the VLDL machinery [48–50]. We first evaluated the compatibility of the RNA ISH protocol with detection of virion associated HCV RNA. Buoyant density purified HCV virions were processed for RNA detection followed by immunostaining for core protein. As shown in S2 Fig., ~60% (+) RNA puncta (red) colocalize with core puncta (green). Thus, single molecule virion RNAs can be detected by the RNA ISH protocol. We then quantified (+) and (−) strand RNA colocalization with HCV core at 24, 48 and 72 hpi (Fig. 6A,B). As expected, we observed (+) strand colocalization with core that increases with time. ~15% of (+) RNA is colocalized with core at 24 hpi, which increases to ~35% at 72 hpi. We did observe some (−) strand colocalization with core; however it did not increase over the time course, suggesting that it was unrelated to virion assembly. An intensity line profile (Fig. 6C) showed that the (−) strand (magenta) signal, although “colocalized” does not identically overlap with LD-associated core, but is instead juxtaposed to the LD-associated core. In contrast, the (+) strand (red peak) overlaps the core (green) peak suggesting full colocalization. Thus, residual localization of (−) RNA with core likely reflects the close proximity of sites of replication and assembly.

To quantify intracellular assembled virions we made use of an antibody (CBH-5) that recognizes E2 on fully assembled virions [51]. This E2 localization pattern is very different from the pattern of E2 localization using E2 antibodies that recognize ER localized E2 (S3 Fig.). Furthermore, CBH-5 staining in an established HCV assembly mutant (NS2-G10P) [52] is at background levels similar to an uninfected control (S4 Fig.). At 48 and 72 hpi, approximately 10% of (+) strand puncta colocalize with E2 (Fig. 6D,E). We observe that virtually all of the E2 puncta (green) colocalize with (+) strand RNAs (red), while minimal colocalization of E2 was observed with (−) strand RNAs (magenta).

### Kinetic analysis of genomic HCV RNA fate

**Fig 7:**
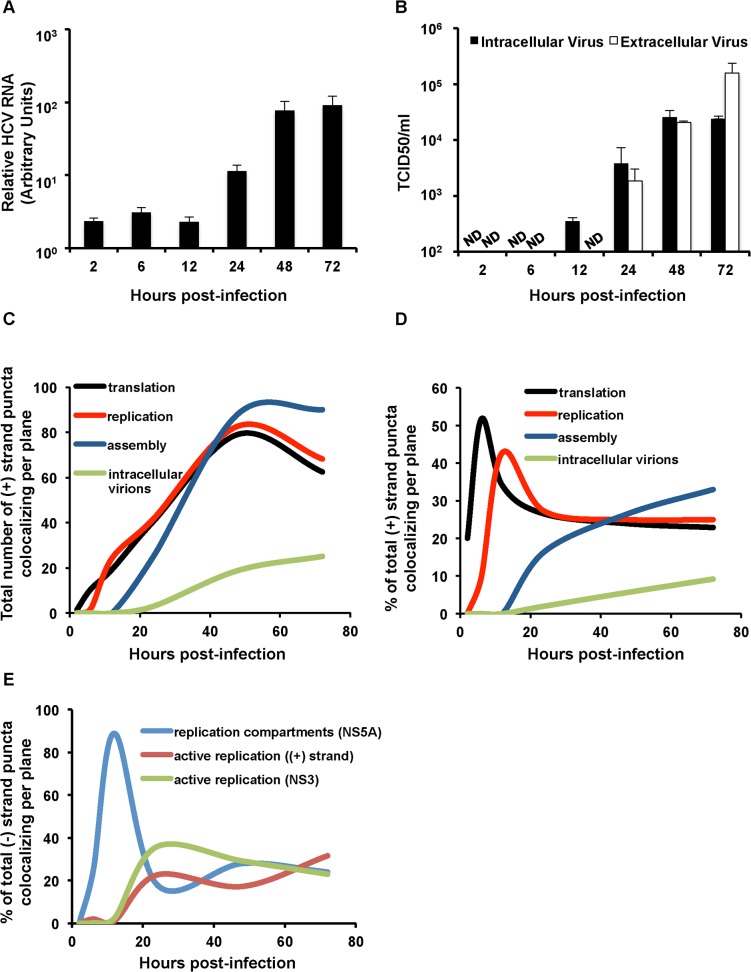
Kinetic analysis of genomic RNA fate. **A**. Huh-7.5 cells were infected with HCV at MOI = 1.5. RNA was collected at the indicated time points post-infection and quantified with real-time RT-PCR. Error bar, standard deviation. **B**. Intra- and extra-cellular viral supernatants from infections in panel A were collected at the indicated time points and titered by limiting dilution assay. Shown are the averages of two sets of titer data. Error bar, standard deviation. **C**. Total (+) puncta or **D**. Percent of (+) strand puncta colocalizing with translation (puromycylated ribosomes), replication (NS5A + NS3), assembly (core) and virion (E2) markers over the indicated time course (Figs. 3–6) were plotted using the smooth graph function in Microsoft Excel. **E**. Percent of (−) strand puncta colocalizing with active replication compartments (NS5A), active replication ((+) strands) and active replication (NS3) over the indicated time course plotted using the smooth graph function in Microsoft Excel.

We next plotted the kinetics of the HCV life cycle based on quantitation of the localization of HCV RNAs with markers of translation ((+) strands with puromycylated ribosomes), replication compartment formation ((−) RNA localized with NS5A), active replication (localization of (−) RNA with (+) RNA and NS3), and assembly and intracellular virions ((+) RNA colocalization with core and virion E2, respectively). The standard quantitation of HCV RNA by quantitative RT-PCR (Fig. 7A) and infectious HCV (Fig. 7B) show a typical increase of both over time. HCV RNA increased at 12–24 hpi and plateaued between 48 and 72 hpi, while intracellular virions peaked between 24 and 48 hpi and plateaued by 72 hpi. We first quantified the total (+) RNAs devoted to translation, replication, assembly, or virions as determined in our previous assays (Fig. 7C). We observed similar kinetics of (+) RNA accumulation as compared to the qRT-PCR assay. HCV (+) RNAs were initially associated with translation, while (+) RNAs associated with replication increased from 12 to 48 hpi and then slightly decreased. HCV RNAs associated with virion assembly increased between 24 and 48 hpi and plateaued.

We then quantified the relative amounts of (+) RNA devoted to each process (Fig. 7D). (+) strand RNAs display a defined temporal kinetics, with the majority of (+) RNAs associated with actively translating ribosomes at early times of infection, followed by a peak of replication between 12 and 24 hpi, followed by virion assembly and detection of assembled virions in the cell cytoplasm. Interestingly, after the peaks of (+) RNA association with translation and then replication were displaced, both populations achieved a steady state level of ~25% of total (+) RNAs each, which slowly decayed over time as the levels of virion-associated (+) RNAs increased. In comparing the relative kinetics of each process in the viral life cycle, the slowest kinetic delay is from HCV assembly to accumulation of intracellular virions (Fig. 7D).

Quantitation of HCV (−) RNA colocalization with markers of replication revealed distinct phases of HCV replication (Fig. 7E). Transient active replication could be observed at early time points (4 hpi), however, robust active replication did not occur until between 12 and 24 hpi. This was preceded by a high level of colocalization of NS5A with (−) RNA. These data are consistent with a model wherein low levels of HCV RNA replication precede replication compartment (or membranous web) formation, replication compartments are then formed at 12 hpi, which is then followed by robust HCV RNA replication. The percent of HCV (−) RNAs engaged in active RNA replication then remains relatively constant throughout the time course of infection at ~35%.

## Discussion

Recent improvements in multiplexed single molecule RNA FISH enable sensitive single cell detection of viral RNAs in infected cells. This approach has been used to detect RNA from a number of viruses, including the (+) strand RNA of HCV *in vitro* and *in vivo* [29,53]. In this study, we developed assays for the specific co-detection of HCV (+) and (−) RNA in the same infected cell, in combination with high-resolution microscopy to quantify the kinetics of HCV RNAs at the single cell level. We verified the specificity of these assays. (+) RNA detection was specific to HCV infected- or transfected-cells. RNA puncta increased in abundance during infection, which was precluded by HCV inhibitors. HCV (−) RNA detection had similar properties, in addition to being specific for cells transfected with wild type, but not polymerase defective, HCV RNAs (Fig. 1 and S1 Fig.). We confirmed that this approach has single molecule sensitivity and that the fixation/detection method is compatible with detection of HCV RNAs in infectious virions (S2 Fig.).

We then used this assay to perform a kinetic analysis of HCV infection at the single cell level (Fig. 2). HCV (+) RNA was readily detected at 2 hpi, while the (−) RNA was detected at 6 hpi. Both (+) and (−) RNA accumulation increased over a 48 hour time course, as would be expected. There was considerable cell-to-cell variability in both (+) and (−) RNA accumulation with both showing an ~10-fold range in values at a given time point. This variability was also reflected in (+)/(−) RNA ratio, which ranged from less than one to greater than 35 depending on the infected cell. This large variability in (+)/(−) ratio suggests that distinct host factors that differentially regulate (+) and (−) RNA synthesis are limiting in individual Huh-7.5 cells. Despite this cell-to-cell variability, the mean (+)/(−) RNA ratio of ~10 was relatively constant and is consistent with previous estimates using strand-specific northern blot analysis of HCV replicon cells [3,35] as well as qRT-PCR analysis of HCV-infected whole cell lysates [26].

We next developed microscopic assays to identify HCV RNAs associated with actively translating ribosomes, replication complexes, nucleocapsid assembly, and intra-cellular virions. The colocalization of HCV (+) RNAs with puromycylated ribosomes indicated actively translating HCV RNAs. At 6 hpi, the majority of (+) RNAs were associated with translating ribosomes, and this association subsequently decreased to a steady state of ~30% of total HCV RNAs over the time course of infection (Fig. 3). Interestingly, there was also a high level of colocalization of puromycylated ribosomes with HCV (−) RNAs. While it is possible that HCV (−) RNAs are associated with ribosomes, a more likely interpretation is that sites of HCV translation and replication are in close proximity. (+) RNAs cannot be simultaneously translated and replicated due to steric hindrance between ribosomes traveling 5’ to 3’ and the HCV replicase moving 3’ to 5’ [13]. Models for the differential regulation of HCV translation and replication include spatial separation (e.g. the exclusion of ribosomes from replication compartments) and/or the differential regulation of these processes via protein-RNA and RNA-RNA interactions. The viral polymerase NS5B can only initiate RNA synthesis from the same RNA that it has been translated from (a cis requirement) [54]. Additionally, viral translation is dependent on active RNA replication [55], suggesting that the translation complexes have to be in very close proximity to the replication complexes. In this case, the decision whether to translate or replicate the viral RNA may be modulated by regulation of HCV RNA-RNA or RNA-protein interactions. For example, the HCV RNA kissing loop interaction between the HCV cis-acting replication element and the 3’UTR by EWSR1 may promote a switch between HCV translation and replication [14]. Our data suggest that replication and translation are spatially linked. Development of puromycylation assays compatible with immuno-EM may answer the question of whether active translation occurs within, or adjacent to, HCV replication compartments.

Detection of the (−) RNA, the replication intermediate, is indicative of a viral replication complex. We detect (−) RNA as early as 4 hpi in infected cells, some of which is involved in active replication, as defined by (+) RNA colocalization (Fig. 4). By 12 hpi the majority of (−) RNA is associated with NS5A, but not NS3 or (+) RNA. NS5A has been implicated in the formation of HCV replication compartments [9], which may explain this selective HCV (−) RNA/NS5A colocalization at 12 hpi. We also observe high levels of colocalization of (−) RNA at the 6 hpi time point with large globular fluorescent labeling of ER markers, including ribosomes and calnexin, in a subset of cells (Fig. 3B,D). Given the kinetic coincidence between (−) RNA appearance, NS5A association, and altered ER morphology, it is possible that this represents large scale alterations of the ER associated with replication complex formation. Alternatively, it may be a stress response to infection, such as induction of the ER stress response.

The appearance of (−) RNA and low levels of transient dsRNA replication intermediates at 4 hpi appears to precede HCV replication compartment/membranous web formation, as defined by extensive (−) RNA/NS5A colocalization at 12 hpi, and robust HCV RNA replication (12–24 hpi). The proposed kinetics of membranous web formation is consistent with previous EM data that described the initial appearance of intracellular double membrane vesicles at 16 hpi and further accumulation until 24 hpi [9]. The transient HCV replication prior to replication compartment formation suggests a strategy wherein HCV synthesizes limited amounts of (+) and (−) RNAs early during infection to decrease its reliance in the integrity of the initially infecting HCV (+) RNA. Any damage or modifications to the initial (+) RNA would result in an abortive infection, this it makes sense that the virus would synthesize a small pool of viral RNAs early to maximize the probability of a productive infection.

It is unclear whether these early replication intermediates (4 hpi) are shielded by a small amount of membrane remodeling that precedes the robust replication compartment formation at 12 hpi. If these replication intermediates are not membrane-protected, they may be vulnerable to cytosolic RNA sensors, such RIG-I-like RNA sensors and dsRNA activated protein kinase R (PKR). It remains to be determined whether these RNAs are recognized by innate immune sensors or alternatively, whether HCV has evolved a mechanism to protect these RNAs from detection. Future studies will examine the localization of innate immune RNA sensors with HCV (+), (−), and dsRNAs.

Higher proportions of active replication complexes, as defined by colocalization of (−) RNA with either (+) RNA or NS3 are detected later in infection, between 12 and 24 hpi. This time point corresponds to increases in (+) and (−) RNA fluorescent puncta (Fig. 4), in addition to HCV RNA levels by quantitative real time RT-PCR (Fig. 7A). The proportion of HCV replication complexes that are defined as active (% of (−) RNAs that colocalize with NS3 or (+) RNAs) is consistently ~30% after 24 hpi. This is consistent with predictions from cryo-EM analysis of HCV replication complexes, which indicate that some HCV replication compartments are not compatible with active replication complexes [9].

The specific viral (−) strand detection is of practical interest, since despite extensive efforts; there are few good markers of HCV replication complexes for microscopy analysis. Only a small fraction of the NS5B polymerase (<5%) resides inside replication complexes [35] and other replicase proteins (NS3 and NS5A) have multiple localizations beyond the replication complex. Similarly, there are no cellular proteins have been described that are consistently localized solely to replication complexes. We and many other labs have used a dsRNA antibody to detect putative viral RNA replication complexes [37,56]. Although, this antibody recognizes antigen within replication compartments and is specific to infected cells [9], it is possible that the antibody may also detect structured viral RNAs. We attempted to assess the degree of colocalization of the dsRNA antibody with (−) RNA; however, the fixation and permeabilization steps of the two detection methods were not compatible.

HCV (+) RNAs associated with core both in the proximity of lipid droplets, which are putative sites of nucleocapsid assembly, and in discrete puncta, which may represent assembled virions. The percent HCV (+) RNA that colocalized with core increased during the time course from ~15% at 24 hpi to ~35% at 72 hpi (Fig. 6). Similarly the colocalization with (+) RNA and an E2 antibody that preferentially recognizes E2 incorporated into virions increased over the time course from virtually none at 24 hpi to ~12% of (+) RNAs at 72 hpi. This kinetics mirrors the production of infectious HCV (Fig. 7B). We observed a juxtaposition of ~15% of HCV (−) RNAs with core, consistent with the spatial linkage between sites of RNA replication and assembly. No HCV (−) RNA colocalized with virion E2.

The development of assays to define (+) RNAs associated with translation, replication, nucleocapsid assembly, and intra-cellular virions allowed us to quantify the total number of (+) RNAs, in addition to the proportion of (+) RNAs, associated with each process (Fig. 7). We observed an initial association of (+) RNAs with translation, followed rapidly by replication. The number of (+) RNAs associated with translation or replication increased with similar kinetics and abundance until 48 hpi, after which they diminished slightly. Association of (+) RNAs with nucleocapsid assembly was detected at 24 hpi and peaked at 48 hpi, after which it plateaued as the most abundant class of HCV (+) RNAs in infected cells. Putative association of (+) RNAs with intracellular virions was detected at 48 hpi and increased slightly at 72 hpi. Analysis of the proportion of (+) RNAs associated with each process in the viral life cycle revealed a tightly coordinated regulation. An initial peak of HCV translation at 6 hpi was rapidly displaced by HCV replication at 12–24 hpi, after which both maintained a steady state level of ~25% each of total HCV (+) RNAs. The proportion of (+) RNA associated with nucleocapsid assembly began to displace the replication peak at 24 hpi, after which it gradually increased throughout the time course. Finally, ~10% of HCV (+) RNA associated with intracellular virions was detected at 48 hpi and beyond. The slowest kinetic delay that we observed is from HCV assembly to accumulation of intracellular virions (Fig. 7D). This is consistent with the observation that core in HCV JC1-infected cells (similar to the virus used in this study) has reduced localization at LDs as compared with the less efficiently assembled HCV JFH1 virus [57], suggesting that virion nucleocapsid assembly is not a rate-limiting event in core trafficking, but that HCV envelopment may be rate-limiting.

Future studies will investigate viral and cellular factors that regulate the temporal kinetics of (+) RNA fate, and thus the coordinated integration of the viral life cycle.

## Materials and Methods

### Cells and virus

Huh-7.5 cells [58] were grown in Dulbecco’s modified high glucose media (DMEM; Invitrogen) supplemented with 10% fetal bovine serum (FBS; Atlanta Biologicals), nonessential amino acids (NEAA, 0.1mM; Gibco), 1% penicillin-streptomycin (Gibco), and maintained in 5% CO_2_ at 37°C. For viral stock propagation, Huh-7.5 cells were electroporated with HCV genotype 2a infectious clone pJFHxJ6-CNS2C3 RNA [59], which is similar to the HCV JC1 virus [60], and viral supernatants were collected for up to 5 passages of the electroporated cells. Viral titers were determined by limiting dilution and immunohistochemical staining using an antibody directed to NS5A (9E10) [61].

### Pharmacological inhibitors

Drugs were dissolved in DMSO and used at indicated concentrations. Sofosbuvir (PSI-7977) (Medchem Express), 10 μM; Daclatasvir (SelleckChem) 1nM.

### QuantiGene ViewRNA ISH

The QuantiGene ViewRNA ISH Cell Assay kit (QVC0001) and probes were purchased from Affymetrix, CA. The (+) strand probe set (VF1–10121) is designed to anneal to bases 3733–4870 of the HCV JFH1 genome, while the (−) strand probe set (VF6–11102) anneals to the negative strand with corresponding bases 4904–5911 on the positive strand. For RNA ISH, Huh-7.5 cells seeded onto 12 mm round coverslips at a confluency of 60–70% were infected with HCV at a multiplicity of infection (MOI) of 1.5. At indicated times post-infection, coverslips were fixed in 4% paraformaldehyde for 30 minutes and permeabilized with 70% ethanol for 1 hour at 4°C. Subsequently, coverslips were co-incubated with the (+) and (−) HCV specific probes (1:100 in Probe Set Diluent QF) for 3h at 40°C. Washing and signal amplification were performed as per manufacturer’s instructions. Following the last wash, coverslips were blocked with 20% goat serum in PBS followed by overnight incubation with primary antibodies as follows: 1:25000 anti-NS5A 9E10 (a kind gift of Charles Rice, Rockefeller University), 1:200 anti-NS3 (Virogen), 1:1000 anti-calnexin (Enzo Life Sciences), 1:100 anti-core (Virostat), 1:100 anti-E2 CBH-5 (a kind gift from Steven Foung, Stanford University), 1:2000 anti-E2 1C1 (a kind gift from Arash Grakoui, Emory University). Following primary antibody incubation, coverslips were washed two times with PBS and incubated with AlexaFluor 488 conjugated secondary antibody (1:1000) for 1 hour before mounting on Prolong Gold with DAPI reagent (Invitrogen).

### Tyramide signal amplification

The tyramide signal amplification kit (Molecular Probes; T20912) was used as per manufacturer’s instructions. Following QuantiGene ViewRNA ISH labeling, cells in coverslips were incubated in peroxidase quenching buffer for 1 hour to quench endogenous peroxidase activity. Following blocking with 1% provided blocking reagent, coverslips were incubated with primary antibodies (1:25000 anti-NS5A or 1:200 anti-NS3) for 1 hour. Coverslips were washed two times with PBS and then incubated with a 1:150 dilution of the HRP conjugate for 1 hour. Following two washes in PBS, coverslips were labeled with the tyramide working solution for 5 minutes before mounting on Prolong Gold reagent (Invitrogen).

### Puromycylation of active translating ribosomes

Huh-7.5 cells seeded onto poly-lysine treated coverslips were incubated in media supplemented with 91 μM puromycin (Invitrogen) and 208 μM emetine (Sigma) for 5 minutes at 37°C. Cells were then incubated for 2 minutes on ice with permeabilization buffer (50 mM Tris-HCl, pH 7.5, 5 mM MgCl2, 25 mM KCl, 355 μM cycloheximide, EDTA-free protease inhibitors, 10 U/ml RNaseOut and 0.015% digitonin. After this extraction step, cells were washed once with PBS, fixed in 4% paraformaldehyde and processed for QuantiGene ViewRNA ISH. Following RNA labeling and blocking, cells were incubated with the PMY-2A4 monoclonal antibody developed by Jonathan Yewdell, National Institutes of Health, and obtained from the Developmental Studies Hybridoma Bank, University of Iowa.

### Immunofluorescence microscopy

Coverslips were imaged for (+) strand HCV RNAs by using the PMT detector set for the 566–608 nm wavelength range, (−) strand by using the HyD detector set for the 650–714 nm wavelength range, AlexaFluor 488 was visualized by using the HyD detector set for the 495–535 nm wavelength range, and DAPI stained nuclei were visualized by using the PMT detector set for the 424–465 nm wavelength range. Immunofluorescence images were taken using a Leica SP5 II AOBS Tandem Scanner Spectral confocal microscope with a 100X 1.46 oil objective. Post-acquisition, images were processed using Fiji (Image J) software. Specifically, each red, green, magenta and blue stack was separated into individual channels. Each channel was processed by using the Filters/Unsharp Mask tool and then merged into a single image. Colocalization was determined by using the JACoP plugin post image threshold. The Manders’ coefficients were converted to % colocalization. Each graph data point represents the average of 25 images (single cells) and depending on the time point the average total number of (−) strand puncta analyzed ranges from ~25 to ~2500 while the total number of (+) strand puncta analyzed ranges from ~25 to ~10000.

### Quantitative real time RT-PCR

At indicated times post-infection, cells were washed twice with PBS and lysed in RLT lysis buffer (Qiagen). RNA was isolated using the RNAeasy kit (Qiagen). HCV RNA levels were analyzed using the Platinum qRT-PCR Thermoscript One-Step System (Applied Biosystems) and a custom designed primer probe set: Forward: 5’-ACTTCATTAGCGGCATCCAATAC, Reverse: 5’-CGGCACTGAATGCCATCAT, Probe: 5’-6FAM-CAGGATTGTCAACACTGCCAGGGAACC-Iowa Black. RNA was reverse transcribed for 30 min at 50°C followed by an inactivation step (95°C for 6 min). The cDNA was then amplified for 50 cycles of 95°C for 15 seconds, 60°C for 1 min. HCV RNA levels were normalized to 18S ribosomal RNA. All assays were performed on an ABI 7300 system and analyzed with SDS 1.3 software (Applied Biosystems).

### Statistical analysis

T-test was performed to compare data sets. P-values less than 0.05 were considered significant.

## Supporting Information

S1 Fig Strand specific accumulation of HCV RNAs in response to anti-HCV drugs. **A**. Huh-7.5 cells were infected with HCV and treated with DMSO, Sofosbuvir (10 μM), or Daclatasvir (1nM). Cells were fixed at 6 and 48 hpi and processed for strand specific RNA detection. Scale bar is 5 μm. **B**. Quantification of images in panel A. Error bars represent standard deviation from 25 different images. * p< 0.05, **p<0.005.
(TIF)

S2 Fig Detection of (+) strand RNA and core in extracellular virions. **A**. Purified virions from a sucrose gradient were aliquoted onto poly-lysine coverslips, fixed with 4% paraformaldehyde, permeabilized with 70% ethanol and subjected to (+) strand RNA detection followed by immunofluorescence for core protein. **B**. Quantification of images shown in panel A.
(TIF)

S3 Fig Staining patterns of HCV E2. Huh-7.5 cells were infected with HCV at MOI = 1.5, fixed at 24, 48, and 72 hpi and processed for strand specific RNA detection followed by immunofluorescence staining for E2 using antibody 1C1 (1:2000). Scale bar is 5 μm.
(TIF)

S4 Fig CBH-5 staining in an assembly deficient mutant. **A**. Huh-7.5 cells were electroporated with the indicated HCV RNA constructs and cells were fixed and processed for RNA detection at 4 days post-electroporation. Immunofluorescence staining for virion E2 was performed using CBH-5 antibody. **B**. Quantification of images shown in panel A using GraphPad Prism software, ***p<0.0001.
(TIF)

S1 Movie 3D reconstruction of Z-stacks across infected cell. Infected Huh-7.5 cells were fixed at 48 hpi and processed for strand specific RNA detection. Shown are 32 slices 0.08 μm apart across the cell that were 3D reconstituted using Stacks in ImageJ.
(AVI)
