# Supplementary figures and images for: Spatiotemporal Analysis of Hepatitis C Virus Infection
Source: PLoS Pathog. 2015 Mar 30;11(3):e1004758. doi: 10.1371/journal.ppat.1004758 (PMC4378894; doi:10.1371/journal.ppat.1004758)

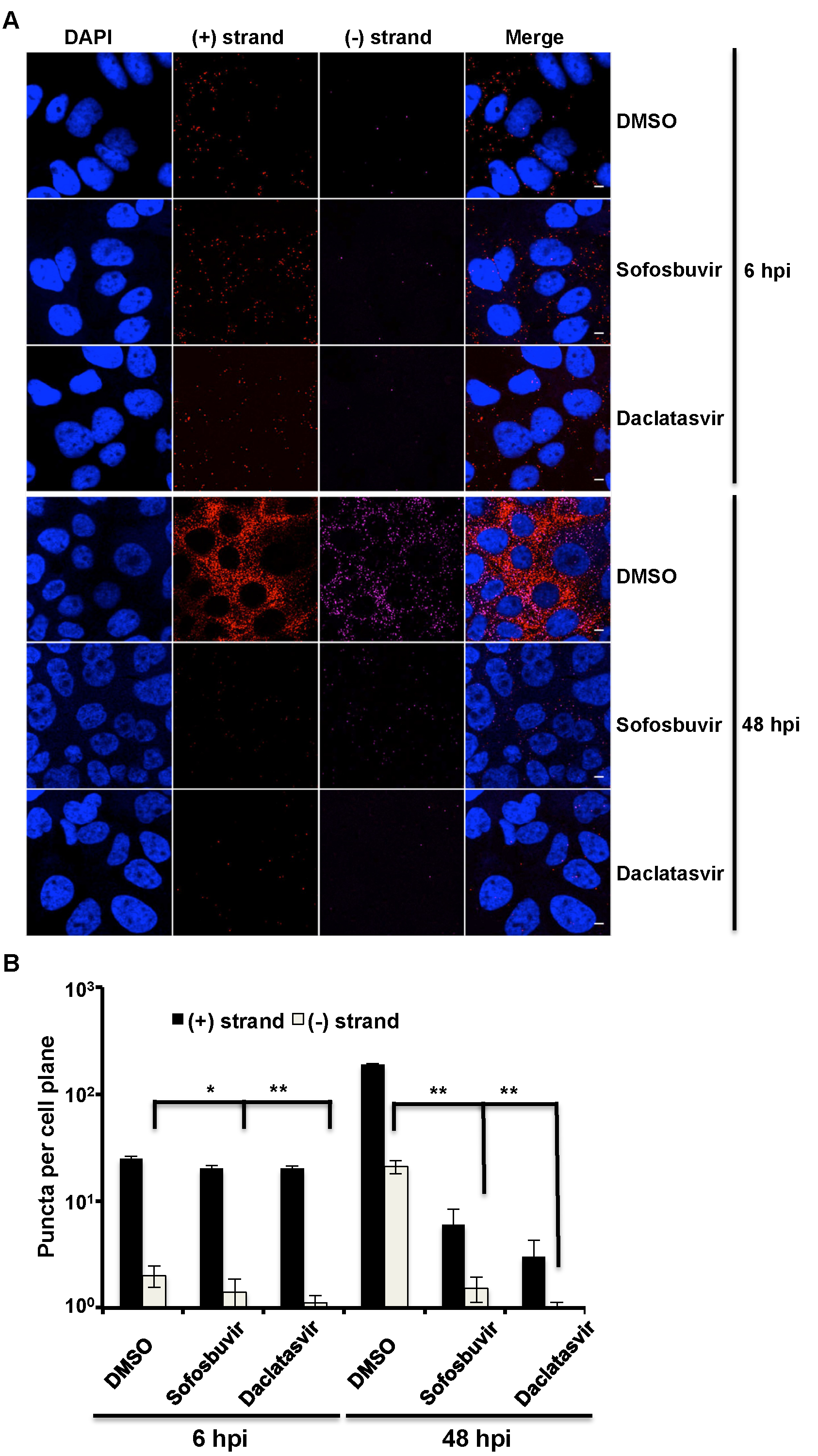

Supplement: S1 Fig — A. Huh-7.5 cells were infected with HCV and treated with DMSO, Sofosbuvir (10 μM), or Daclatasvir (1nM). Cells were fixed at 6 and 48 hpi and processed for strand specific RNA detection. Scale bar is 5 μm. B. Quantification of images in panel A. Error bars represent standard deviation from 25 different images. * p< 0.05, **p<0.005. (TIF) [file ppat.1004758.s001.tif]

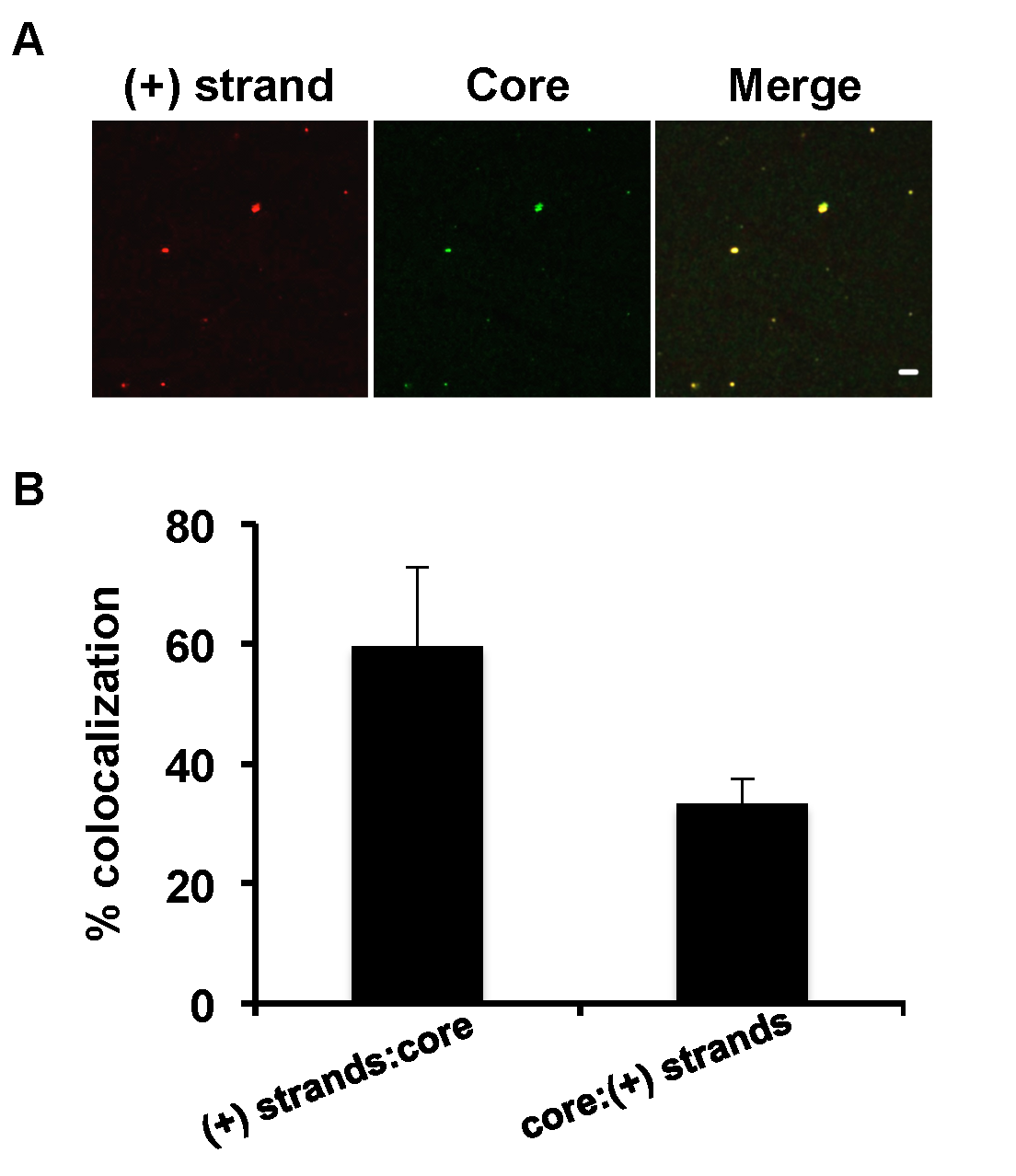

Supplement: S2 Fig — A. Purified virions from a sucrose gradient were aliquoted onto poly-lysine coverslips, fixed with 4% paraformaldehyde, permeabilized with 70% ethanol and subjected to (+) strand RNA detection followed by immunofluorescence for core protein. B. Quantification of images shown in panel A. (TIF) [file ppat.1004758.s002.tif]

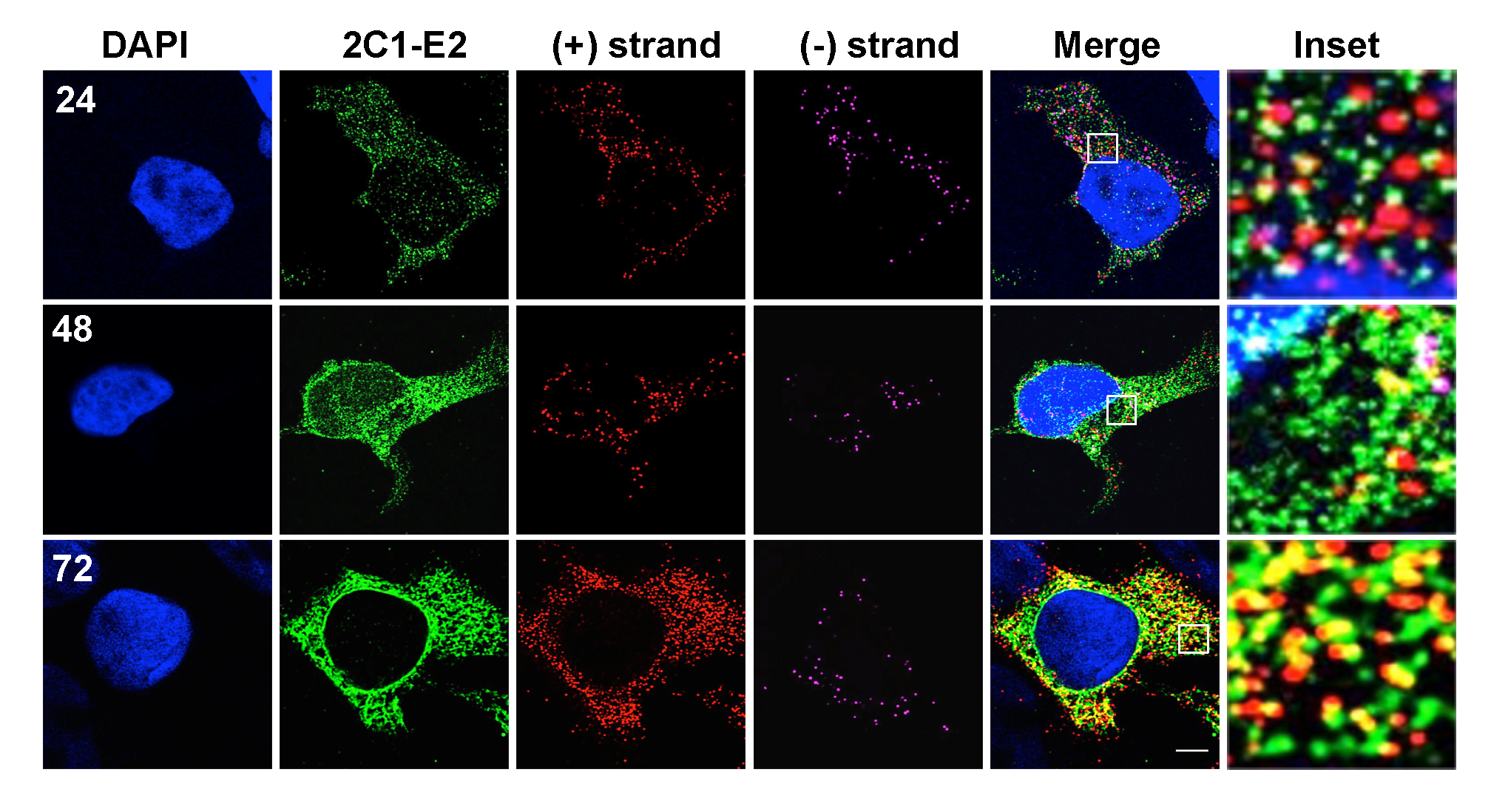

Supplement: S3 Fig — Huh-7.5 cells were infected with HCV at MOI = 1.5, fixed at 24, 48, and 72 hpi and processed for strand specific RNA detection followed by immunofluorescence staining for E2 using antibody 1C1 (1:2000). Scale bar is 5 μm. (TIF) [file ppat.1004758.s003.tif]

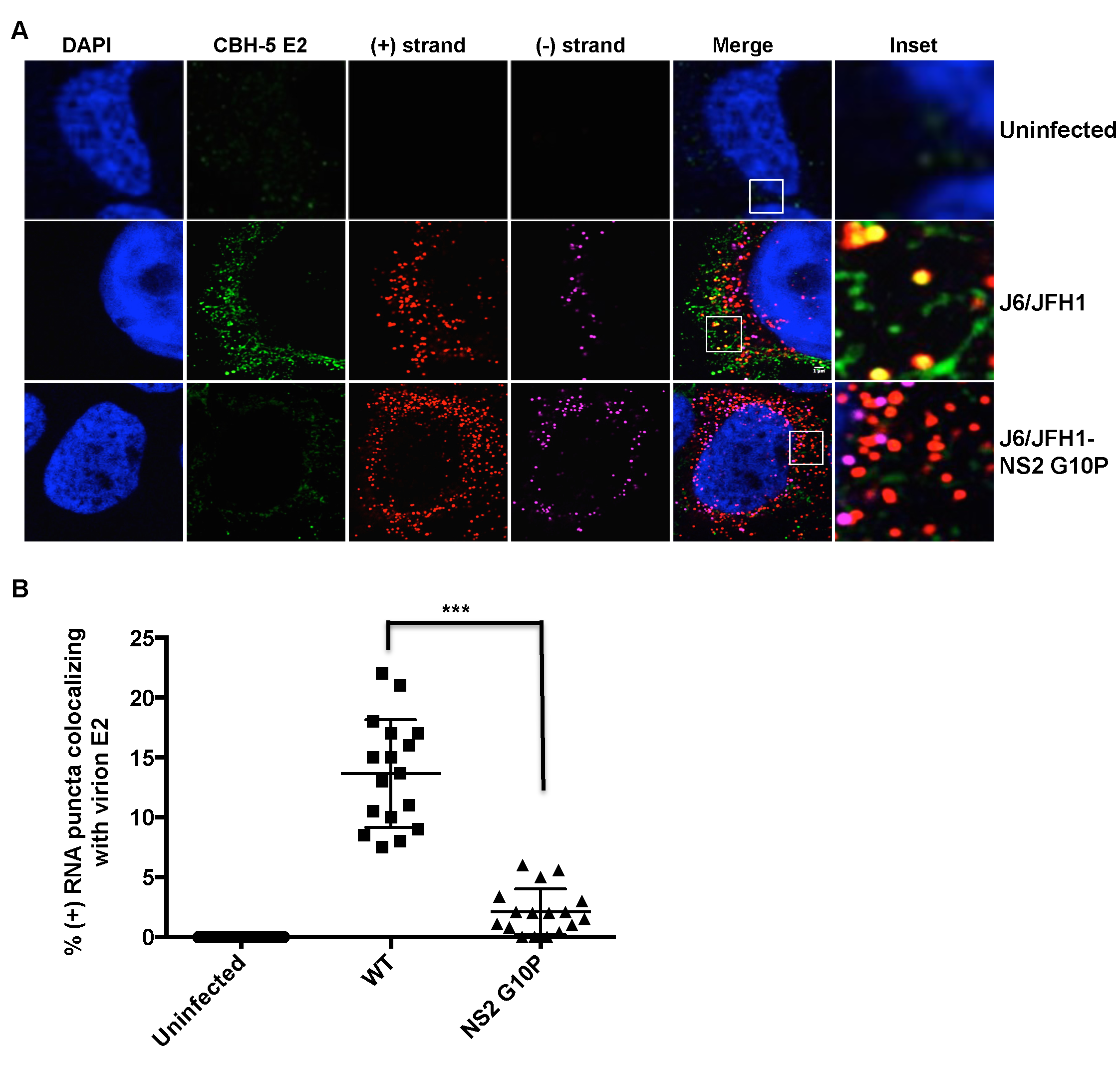

Supplement: S4 Fig — A. Huh-7.5 cells were electroporated with the indicated HCV RNA constructs and cells were fixed and processed for RNA detection at 4 days post-electroporation. Immunofluorescence staining for virion E2 was performed using CBH-5 antibody. B. Quantification of images shown in panel A using GraphPad Prism software, ***p<0.0001. (TIF) [file ppat.1004758.s004.tif]
